# Python-assisted biological knowledge acquisition method to trigger design inspiration

**DOI:** 10.1038/s41598-022-11833-1

**Published:** 2022-05-12

**Authors:** Z. M. Zha, H. Zhang, G. A. Aggidis

**Affiliations:** 1 Ocean Engineering Equipment College, Zhejiang Ocean University, Zhoushan, 316022 Zhejiang People’s Republic of China; 2 Engineering Department, Lancaster University Renewable Energy Group and Fluid Machinery Group, Lancaster, LA14YW UK

**Keywords:** Plant sciences, Zoology, Materials science

## Abstract

Design inspiration comes from the continuous stimulation of external information and the continuous accumulation of knowledge. In order to obtain an ideal design inspiration from nature, researchers have proposed a large number of biological information retrieval and knowledge acquisition methods. But how to purposefully acquire valuable biological knowledge in order to effectively stimulate design inspiration and produce the novel and feasible designs idea is still an urgent problem to be solved. This paper proposes a method for acquiring valuable biological knowledge to efficiently stimulate inspiration and quickly conceive solutions in engineering design. First, keywords, such as the functional requirements and key components of design objects, are selected as the engineering terminologies. Next, biological keywords related to the engineering terminologies are searched from the biological dictionary and biology websites. Then in order to retrieve enough biological knowledge, these biological keywords are expanded manually and automatically respectively based on Thesaurus Webpage and WordNet database, and expanded keywords are filtered according to repeated words and different forms of the same words. Finally, in the biological knowledge base, biological keywords that had been filtered are used to obtain biological knowledge with Python web crawler programming. Through an example of application for ship equipment, the effectiveness of the method is verified.

## Introduction

Most creative work requires inspiration to stimulate imagination to solve difficult problems. Inspiration is a kind of non-logical thinking, which not only comes from the stimulation of external conditions, but also comes from the accumulation of knowledge^1^. Knowledge has many forms, such as design knowledge and biological knowledge. Biological knowledge contains more rare and delicate structures, so it can trigger more inspiration. Most of the existing biological knowledge and information carriers are websites, texts, and biological dictionaries. Many researchers acquire some knowledge, such as biological principles, shapes, functions, and their own unique attributes, to stimulate design innovation from these carriers^2^. Some biological mechanisms are directly imitated, while others tend to inspire the ideas of design^3^. Therefore, acquiring a wealth of biological knowledge is very important for stimulating design inspiration.

Biological knowledge can be obtained from different information carriers, such as books, papers, databases, corpora, biological websites, etc.^4–10^. For different carriers, the methods of obtaining them are different. For example, Shu et al.^11,12^ used a vocabulary language framework to systematically map engineering terms to biologically meaningful keywords, and then used automated search tools to obtain biological knowledge from various biological resources such as books and papers in natural language. Nagel et al.^13^ used the Engineering Biology (E2B) vocabulary to link biological functions and processes with engineering terms, combined with concept generation software models (MEMIC), and obtained biological knowledge by searching biological knowledge bases and engineering knowledge bases. Most of these methods are suitable for designers with a certain biological background or require biologists and engineers to collaborate to complete knowledge transfer and exchange. Therefore, for designers who lack biological background, the acquisition and application of biological knowledge are still the difficult problem^14^. In order to obtain biological knowledge that is more direct and more in line with engineering design goals, this article proposes a method of acquiring biological knowledge for engineers without biological background, which can help them quickly acquire a large amount of valuable biological knowledge.

This paper first extracts engineering keywords according to the purpose functions, means functions and key components of design objects. Next, biological information related to engineering keywords is searched in biological databases such as biological dictionaries and biological web pages, and the biological keywords are extracted from them. Then, these biological words are expanded in two ways. One way is to extract the synonym set of biological words in Thesaurus. Another way is using Python programming to automatically extract the hypernym, hyponyms, and the synonym set of biological words from the WordNet database. After filtering the expanded biological words, some valuable biological keywords are obtained. Finally, based on the obtained words, biological knowledge that can inspire design inspiration is acquired in AskNature with the help of Python programming, and the design inspiration triggered by biological characteristics and phenomenon information is used to solve engineering design problems.

The following section “Acquisition of biological knowledge” introduces the current acquisition methods and applications of biological knowledge. Section “Python-assisted method for acquiring biological knowledge” introduces the detailed process from engineering terms to biological knowledge. Section “An example of application” provides case studies. Section “Discussion” discusses the expansion methods under different biological backgrounds and comparative experiments, and Section "Conclusions" provides the conclusions.

## Acquisition of biological knowledge

In the process of using biological knowledge to inspire design inspiration and solving problems, an important work is to acquire biological knowledge. Many researchers have conducted extensive and in-depth research on this aspect. The research includes not only the methods of obtaining biological knowledge, but also the applications of biological knowledge.

### Methods of obtaining biological knowledge

Valuable biological knowledge is very helpful to stimulate design inspiration. In order to obtain biological knowledge effectively and efficiently, researchers have done a large number of works on the acquisition methods of biological knowledge. Related researches mainly include the following aspects:

- *Selection of search terms* In order to meet the needs of designers, researchers use engineering terms related to product features as search terms. Some researchers chose a single type of engineering term as a search term, such as function^15^, function-flow^16^ or shape^17^ to search for biological solutions. Some researchers chose multiple types of engineering terms as search terms, such as function and effect^18^, function, attribute, and environment^19,20^ to obtain more biological solution. The more types of search terms, the richer the biological information obtained, but how to accurately identify the features of the product and effectively use other features of the product are issues that need to be considered.
- *Expansion of search terms* In order to obtain more search terms for retrieving biological information, researchers have developed a number of dictionaries and thesaurus. For example, the engineering-to-biology thesaurus^21^, Functional Basis^22^ were used to convert engineering terms into biological terms to overcome obstacles caused by different terminologies in different fields. Nagel^23^ have solved the problems of cross-domain design by establishing an engineering-biological vocabulary, but the construction of this kind of database requires too much time. There are also many researchers using WordNet to expand search terms^12,15^. WordNet includes many uncommon words. So, a lot of useless words will be gotten after the expansion. The number of search terms expanded is related to the quantity and quality of the words in these dictionaries or databases.
- *Obtaining of search terms* Many researchers are exploring the application of artificial intelligence technology in obtaining search terms.
  To obtain the valuable search terms, Chen et al.^15^ proposed an algorithm to automatically push biological information search keywords. This method enables designers to obtain the required biological information without biological knowledge reserves. However, the size of the biological corpus will affect the credibility of the pushed keywords. Li^24^ used mining technology to extract keywords from Autohome. The method of word segmentation will affect the calculation results and calculation efficiency.
  To obtain the rich and relevant search terms, Xu^25^ proposed an algorithm for extracting keywords from webpages based on word span. This algorithm can obtain a wealth of search terms, but its accuracy is related to generation method of high frequency words. Liu^26^ proposed a supervised keyword extraction algorithm, which can recognize keywords in the test documents, but the accuracy of recognition will be affected by the amount of labeled training data. Liu^27^ proposed a method to obtain semantic keywords based on the ontology. The method can improve relevance, but the construction of the ontology is more troublesome.
- *Retrieval of biological information* In order to discover biological inspiration, some retrieval systems have been developed, such as Webcrawler^28^, citation cataloguing system^29^, BIOscrabble^19,20^. In addition, Kim et al.^30^ developed a causality-based overall representation framework of biological systems, an ontology-based "fully connected" knowledge base and retrieval system as a knowledge-based recommendation system that supports bionic design. Willocx et al.^31^ used three different methods to find biological inspiration and found that processing retrieved biological articles is still a difficult and time-consuming task. Therefore, biological information retrieval system still needs to be improved to be more friendly to engineers without biological background.

### Application of biological knowledge

Biological knowledge is one of the sources of inspiration for innovation. Some novel designs can be conceived with the help of biological knowledge of shape, function, structure, texture, color, and strategy^3^. The methods of applying biological knowledge have been proposed.

- Design process
  Van et al.^32^ mainly described the design process based on a top-down approach. He used biological phenomena and data for the development and application of products, machine tools, processes and manufacturing systems.
  Goel et al.^33^ analyzed 74 bio-inspired design cases in the library and compared the problem-driven bio-design process with the solution-based design process. It can be discovered that the solution-based process is easier to lead to a multifunctional design than a problem-driven process.
  Lenau et al.^34^ reviewed the characterization and application of design paradigms. According to dominant opportunities, challenges, and knowledge characteristics, different design paradigms can be used to realize the biologically inspired design process.
  Peters^35^ proposed two spiral models of the bionic design process. That is from biology to design and from problems to biology. The top-down bionic design process can effectively find solutions that are conducive to product innovation and design. Extraction and expression of morphological features are targeted more, which can better grasp the development direction of innovative design^36–38^. The bottom-up bionic design process of product shape is more aimed at the extraction and expression of the intrinsic characteristics of biological shape, which is good for grasping the direction of innovative design and quickly find the solution of product innovation. Its direction is more diversified and open, and product design schemes are also more diversified^39^.
- Design method
  TAN et al.^40^ revealed that the biological knowledge applied to the engineering innovation has evolved from imitating simple biological characteristics to building new products, design processes and new manufacturing systems.
  Bogatyrev^41^ constructed a knowledge base of biological effects based on TRIZ theory to assist the bionic design process. TRIZ can be used to solve the contradictions and problems encountered in the bionic design process. It is the key to realizing multi-dimensional (shape, function, etc.) product bionic design.
  Helms^42^ used analogies to develop solutions for engineering problems. Three frequently used arguments are: (a) The mature performance of biological systems; (b) The potential of sustainable products and (c) the potential for finding ready-made solutions. Nature is a largely undeveloped field, so it is still very likely to stimulate the potential for more sustainable and innovative products. At the same time, the biological knowledge can be used to inspire breakthrough innovative ideas^43^, which can be further developed into new patents.
  Through the above analysis of the current research works, it can be found that the limitations of existing methods are as follows: (a) How to realize the conversion of engineering words to valuable biological words; (b) How to expand the search for biological knowledge; (c) How to improve the efficiency of acquiring biological knowledge; and (d) How to transfer the acquired biological knowledge into design and creation. In response to these problems, this paper proposes a Python-based method for acquiring biological knowledge and applies it to the design of a novel bionic winch. Through the design of this winch, the limitations of the prior method are improved. Table 1 shows some biological information retrieval methods that have been developed and researched.

**Table 1:** A comparison of methods of acquiring biological knowledge.

Information carrier		Author	Method	Purpose	Advantage	Disadvantage
Text	AskNature	Wang pan et al.^44^	Proposed web-based automatic information acquisition.	To achieve the combination of portable data and portable code by combining XML and Java.	Combining programming methods to obtain information can automatically save time.	The unified description of biological information in xml limits the inspiration for design.
		Liu Wei et al.^45^	Used multi-biological effects to obtain the relevance between biological knowledge and products	To use the feature clustering principle and the analogy principle to transform the target product and multi-biological effects	It is very innovative to stimulate design inspiration through the influence of nature on living things	The correlation between this kind of biological knowledge and products is difficult to discover
	Relevant corpus text	Mark et al.^46^	Put forward the method of describing biological phenomena to develop concepts and solve a simple problem	The research provided an outline of a strategy that will be applied in the fields of biology and engineering	The biological strategies extracted have provided diverse solutions and inspirations for solving problems.	Inability to transfer information from biology to engineering
	LIFE corpus text	Nagel and Stone^47^	Through an algorithm, utilized the functional basis, Design Repository, MEMIC (Morphological evaluation machine and interactive conceptualizer), organized search tool and engineering to biology thesaurus to create, filter and inspire concept variants	To discover biological inspiration and circumvent the problem of knowledge needs in different fields in the early stages of design,	The computational approach could be used to assist engineering students with discovering the connections between the biology and engineering domains and find innovative solutions to the problem	There are no hyperlinks to add detailed biological information and images to be integrated into the results, and the process takes a long time
Database	The engineering-to-biology thesaurus	Nagel^23^	The engineering-to-biology thesaurus has the potential to aid engineering designers with the comprehension of biological contexts by substituting Functional Basis terms for commonly used biological words	The three key goals of this thesaurus are (1) to lessen the burden when working with knowledge from the biological domain by providing a link between engineering and biological terminologies; (2) to assist designers by establishing connections between the two domains; and (3) to facilitate bioinspired design	The engineering-to-biology thesaurus increases the interaction between the users and the knowledge resource and fosters one to make associations between the engineering and biological lexicons and enhances the designer's ability to use biological information	Search only in text written in natural language format, and cannot be used to search in web-based repositories
	WordNet database	Chen et al.^15^	An algorithm that integrates semantic similarity calculation, data normalization and corpus technology is proposed	To calculate the compound association strength of functional combination words and realize an automatic push of biological information search keywords	The designer obtains the required biological information without the biological knowledge reserve.	The similarity calculation of this algorithm is closely related to WordNet. But WordNet is a vocabulary database which will expand the scope of retrieval
		Chiu et al.^12^	Proposed the natural language processing and computational linguistics for potential semantic retrieval of keywords and related collocation words	Identifying words that frequently collocated with keywords and the relationship between words	Reduce the time to find relevant biological phenomena in natural language	It takes a lot of time to identify the statistics of frequently collocation words

## Python-assisted method for acquiring biological knowledge

Python is an explanatory, interactive and object-oriented cross-platform language with simple syntax and easy reading and writing. It supports both process-oriented programming and object-oriented programming. It has been ported to many platforms and databases. In recent years, with the rise of big data and artificial intelligence technology, Python has been widely used in the fields of web application development, automatic operation and maintenance, web crawling, data analysis, scientific computing, and artificial intelligence. The specific steps of Python-based knowledge acquisition are as following.

### Extracting engineering keywords

When determining the design object and understanding the requirements or purpose of the design, engineering keywords can be extracted first based on the type of knowledge in the product^48^, so that these words are closer to engineering design needs. But keywords have different forms, such as nouns and verbs, which make them have different meanings in a paragraph or text. For example, when “remove” is used as a verb, it means to remove, but when used as a noun, it means distance. Therefore, when keywords are selected, it is necessary to determine the specific meaning and design characteristics and then it can be extracted as an engineering keyword.

### Obtaining biological keywords

After determining the engineering keywords, in order to obtain potential keywords related to biology, it is necessary to search and obtain biological keywords from the biological dictionary or related biological fields. Because biological keywords are directly extracted from biological information, they have a close relationship with biological information. At the same time, these keywords can also increase the understanding of biological information in the later stage, such as capturing pictures and texts about biological knowledge in AskNature.

### Expanding biological keywords

After collecting biological keywords, due to the expanded vocabulary are not comprehensive in the WordNet database, therefore, this paper combines WordNet database and Thesaurus Webpage to expand biological keywords. First, the Thesaurus Webpage is used to expand the synonyms of biological keywords. Then the WordNet database is imported from the NLTK.Corpus database and Python programming is used to automatically query the synonym set, hypernym, and hyponym set of the same part of speech in the vocabulary of the biological keywords. WordNet is like an English dictionary corpus, and it is in the corpora folder under the NLTK data folder. NLTK is easily integrated into the Python environment, so it is easier to use WordNet under the Python environment. At the same time, WordNet is the synset, so it can bring great help when doing cross-language work, but it does not completely belong to any specific field.

### Filtering biological keywords

The expanded keywords that obviously not related to biological information need to be removed to save time of crawling biological information. After deleting irrelevant keywords, the filtered keywords are valuable biological keywords.

### Acquiring biological knowledge

**Figure 1:**
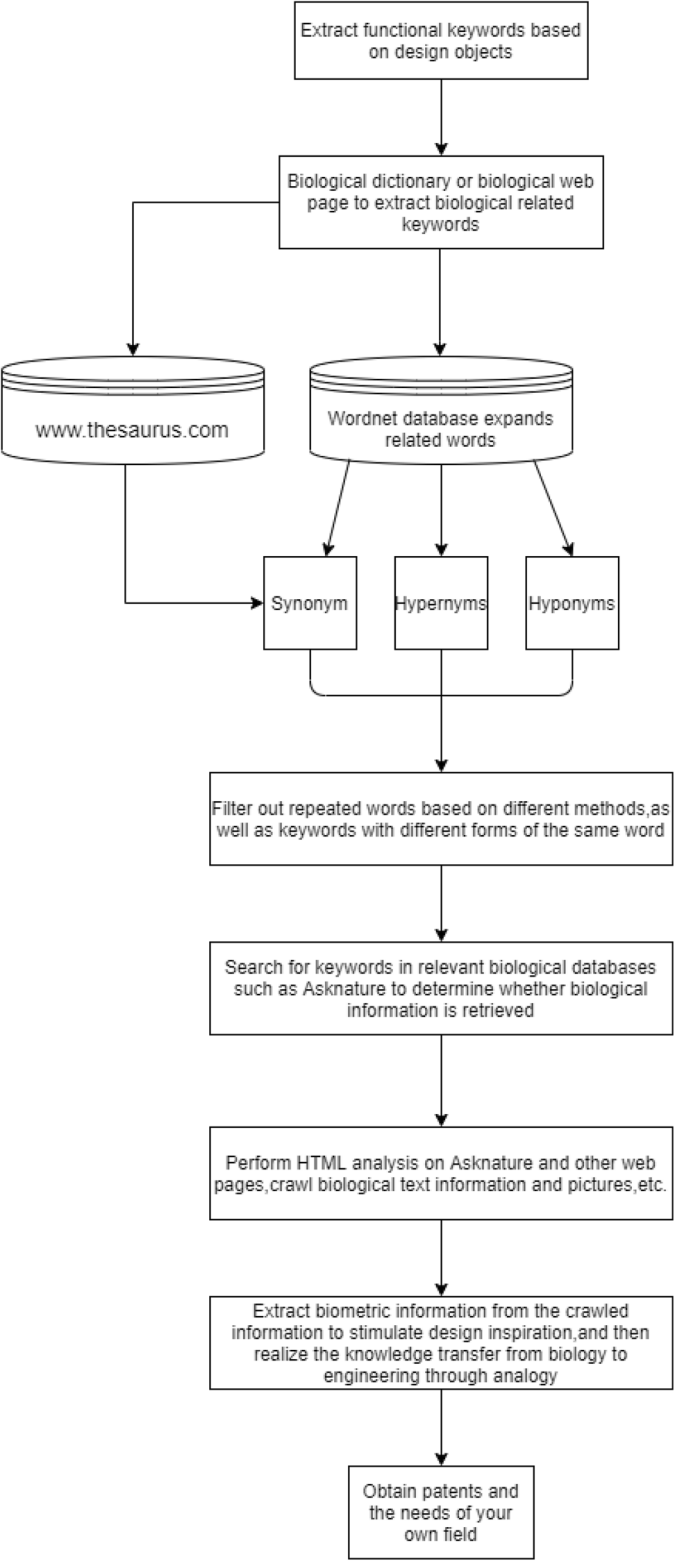
Flow chart of the method of acquiring biological knowledge.

AskNature is a natural science website. When inputting in relevant keywords, we can retrieve the corresponding biological information and knowledge. Using the python crawler can obtain biological phenomena and biological articles with the filtered biological keywords. Thus, biological knowledge obtained will directly affect the thinking of engineering designers and be used to stimulate their design inspiration. The flowchart is shown in Fig. 1.

## An example of application

The figures and plots in this section were drawn by authors using AutoCAD 2017, PowerPoint (version 16.26), or Excel (version 16.26) software.

**Figure 2:**
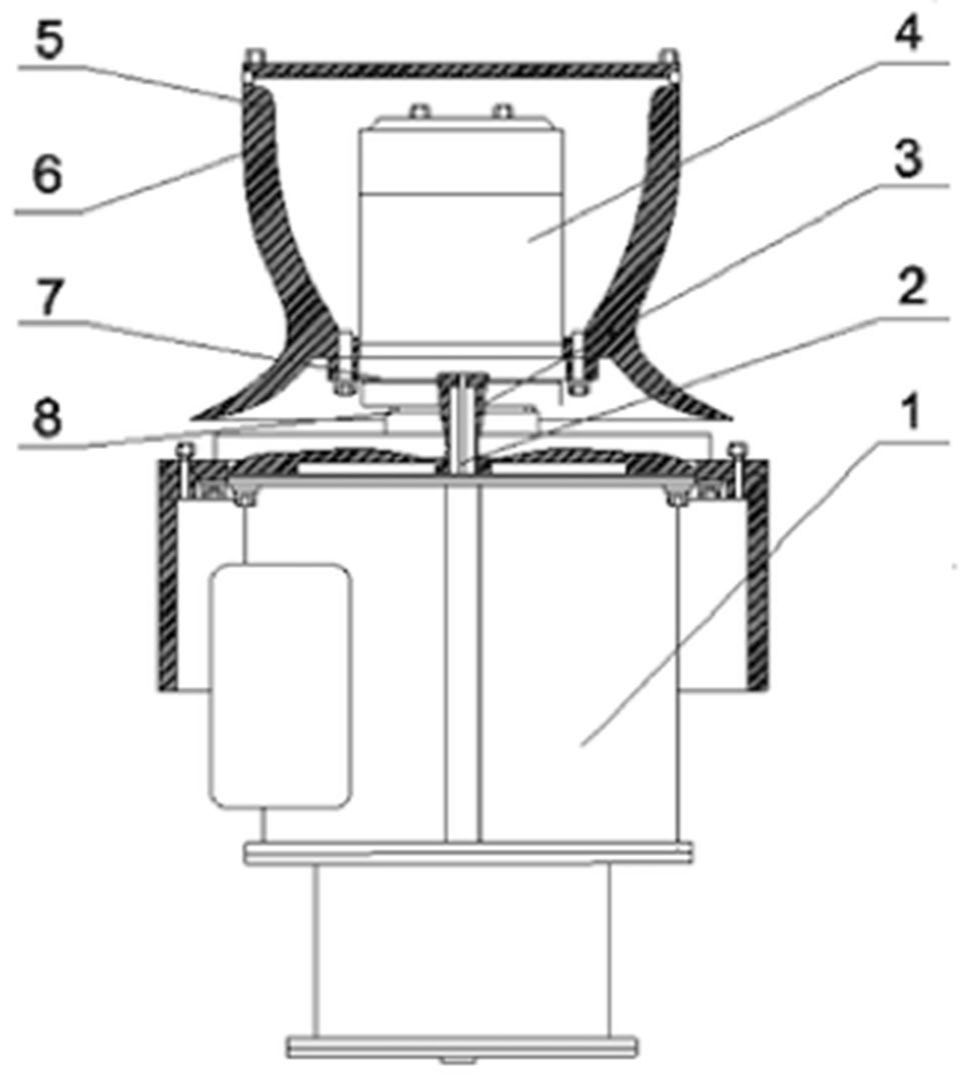
Marine electric mooring winch. 1—Base, 2—Output shaft, 3—Bearing, 4—Rotation shaft, 5—Cable drum shell, 6—Cable drum, 7—Linkage shaft, 8—Cable drum support.

Winch is widely used in various fields, especially ships. Marine winches can be divided into electric winches, hydraulic winches and many other. Among them, the electric winch is usually installed on the main deck at the bow and stern of the ship. It is mainly used for anchoring or retraction of the ship. It is the self-protection and towing device. The marine electric winch is mainly composed of planetary reducer, winch head, main shaft, drive motor and other parts. When working, the power of the motor is transmitted to the rotation of the drum. The rotation of the drum drives the rotation of the driving shaft. The planetary gear reduces the speed of the motor while increasing the output torque to drive the winch to work. The traditional marine winch is shown in Fig. 2.

However, the traditional winch has problems including the following: When the winch stops, the winch will lag behind due to water surface fluctuations. The winch drum will slip when rotating. In addition, the drum will be exposed to external pressure for a long time, which will cause it to wear too much. It also needs to strengthen its pressure resistance, and so on. The following takes a marine winch as an example to introduce the specific implementation steps of the new method proposed in this paper.

### Extracting engineering keywords

This paper aims to improve the above-mentioned marine winches and makes it achieve anti-skid, reduce weight, increase strength and save energy. According to the targets that the electric winch needs to achieve, this article extracts the key components and design purposes of the marine electric winch as engineering keywords, such as lose weight, lightweight design, increase the intensity, non-slip, save electricity, reel, etc. The words usually have polysemy and polymorphism phenomena. So, the meaning of keywords proposed from the design object should be determined.

### Obtaining biological keywords

In order to obtain a large amount of biological knowledge and realize knowledge transfer and biological analogy, this paper discovers potential biological keywords based on the obtained engineering keywords. The biological keywords related to biological language can be found from the dictionary module in the Biology-online.org Webpage^49^, Henderson's Dictionary of Biology^50^, and the biology subject in the module dict.cnki.net^51^.

**Figure 3:**
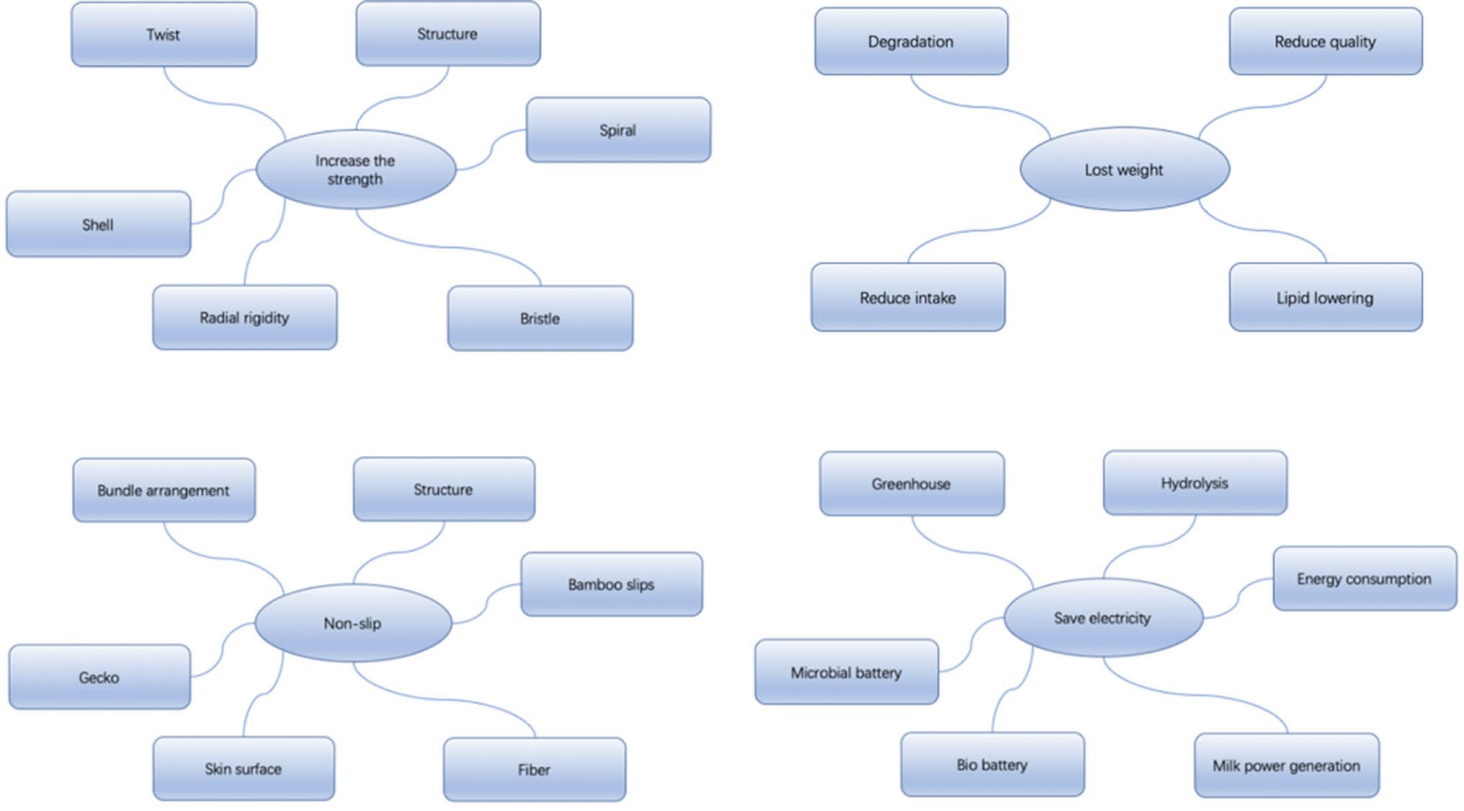
Obtained biological keywords.

The above collected potential keywords are shown in Fig. 3. They are obtained by three different methods and are related to some organisms.

### Expanding biological keywords

**Figure 4:**
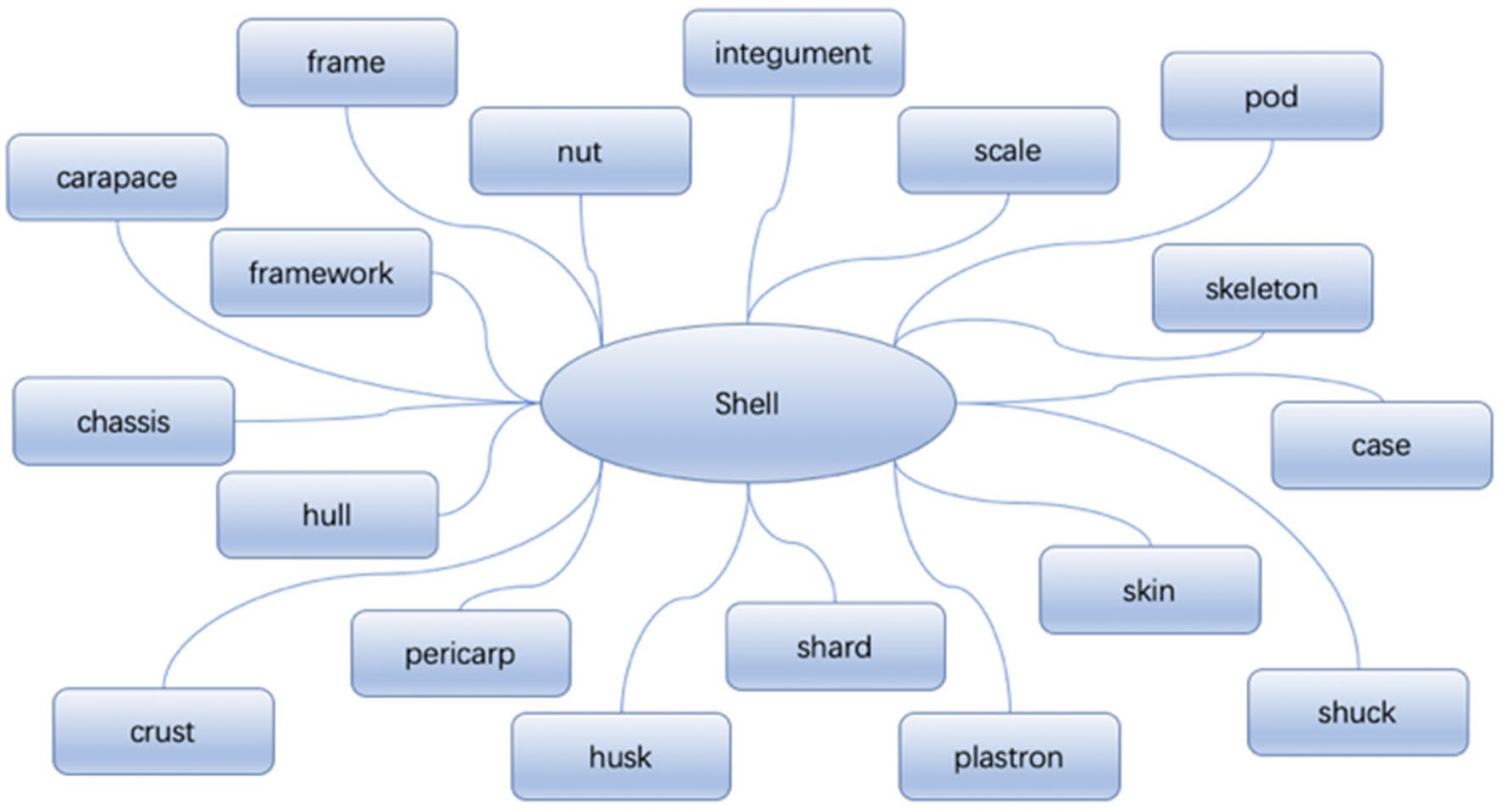
Expanded biological keywords retrieved by the Thesaurus webpage^52^.

To acquire more biological information, the biological keywords need to be expanded by hypernym, a hyponym, and synonyms. In many cases, different words with the same meaning may obtain different information in a biological context. This expansion can supplement the more biological knowledge and information from different angles. At the same time, the expansion of the upper and lower words is also a supplement to the biological knowledge. Therefore, based on the obtained biological keywords, an extended supplement is made in the Python and Thesaurus Webpages respectively. For example, some biological keywords, such as fiber, strength, and shell, are used to expand on the Thesaurus webpage first, and the synonym search results of keyword ‘shell’ are shown in Fig. 4.

Then based on the WordNet database, the biological keywords are segmented and expanded for synonyms, hypernyms, and hyponyms by Python programming. For example, the word ‘reduction’ is expanded on Table 2.

**Table 2:** The expansion of synonyms, hypernyms and hyponyms based on WordNet.

The query for the set of synonyms of the same part of speech of ‘reduction’ is coded as: from nltk.corpus import wordnet as wn sets = wn.synsets('reduction', pos = wn.NOUN) print(sets) The result of the operation:[Synset('decrease.n.04')]	
The query for the set of hypernyms of ‘reduction’ is coded as: from nltk.corpus import wordnet as wn reduction = wn.synset('reduction.n.01') hypernym_sets = reduction.hypernyms() print(hypernym_sets) root_hypernym = reduction.root_hypernyms() print(root_hypernym) The result of the operation:[Synset('change_of_magnitude.n.01')][Synset('entity.n.01')]	
The query for the set of hyponyms of ‘reduction’ is coded as: from nltk.corpus import wordnet as wn reduction = wn.synset('reduction.n.01') hyponym_sets = reduction.hyponyms() print(hyponym_sets) The result of the operation: [Synset('amortization.n.01'), Synset('contraction.n.04'), Synset('cut.n.19'), Synset('cutback.n.01'), Synset('de-escalation.n.01'), Synset('declassification.n.01'), Synset('deflation.n.03'), Synset('depletion.n.01'), Synset('depreciation.n.01'), Synset('devaluation.n.02'), Synset('devitalization.n.01'), Synset('discount.n.01'), Synset('easing.n.02'), Synset('extenuation.n.02'), Synset('lowering.n.01'), Synset('minimization.n.01'), Synset('moderation.n.04'), Synset('reverse_split.n.01'), Synset('rollback.n.02'), Synset('shortening.n.02'), Synset('shrinking.n.02'), Synset('subtraction.n.02'), Synset('tax_credit.n.01'), Synset('tax_shelter.n.01'), Synset('weakening.n.02')]	

**Figure 5:**
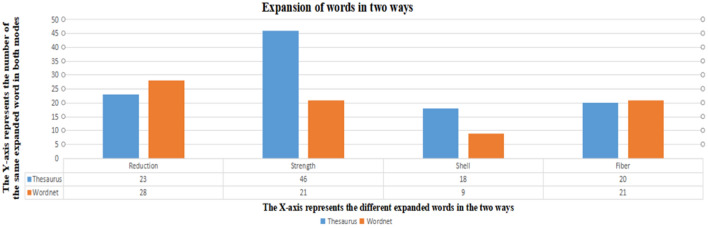
The number of words expanded by the two ways.

In Fig. 5, the two expansion methods are compared based on the number of expanded four biological terminologies. It is found that the words based on WordNet expansion do not necessarily exist in the web page. Therefore, after the expansion of biological terms in two ways, the number of words used to retrieve biological information will increase significantly. At the same time, the number of retrieved biometric information will increase.

### Filtering biological keywords

There are too many words obtained through web search and Python programming. In order to obtain more valuable biological information in a short time, it is necessary to filter the expanded biological keywords. The basis of filtering is: (a) The repeated words obtained by two ways; (b) Different forms of the same word in two ways. The filtered biological keywords are shown on Table 3.

**Table 3:** Biological keywords are filtered by instance abbreviations.

Reduction	Contraction, rebate, decrease, discount, abatement, lowering, depreciation, depletion, easing, degradation, shortening, deflation, reverse

### Acquiring biological knowledge

#### Collection of biological information based on AskNature

**Figure 6:**
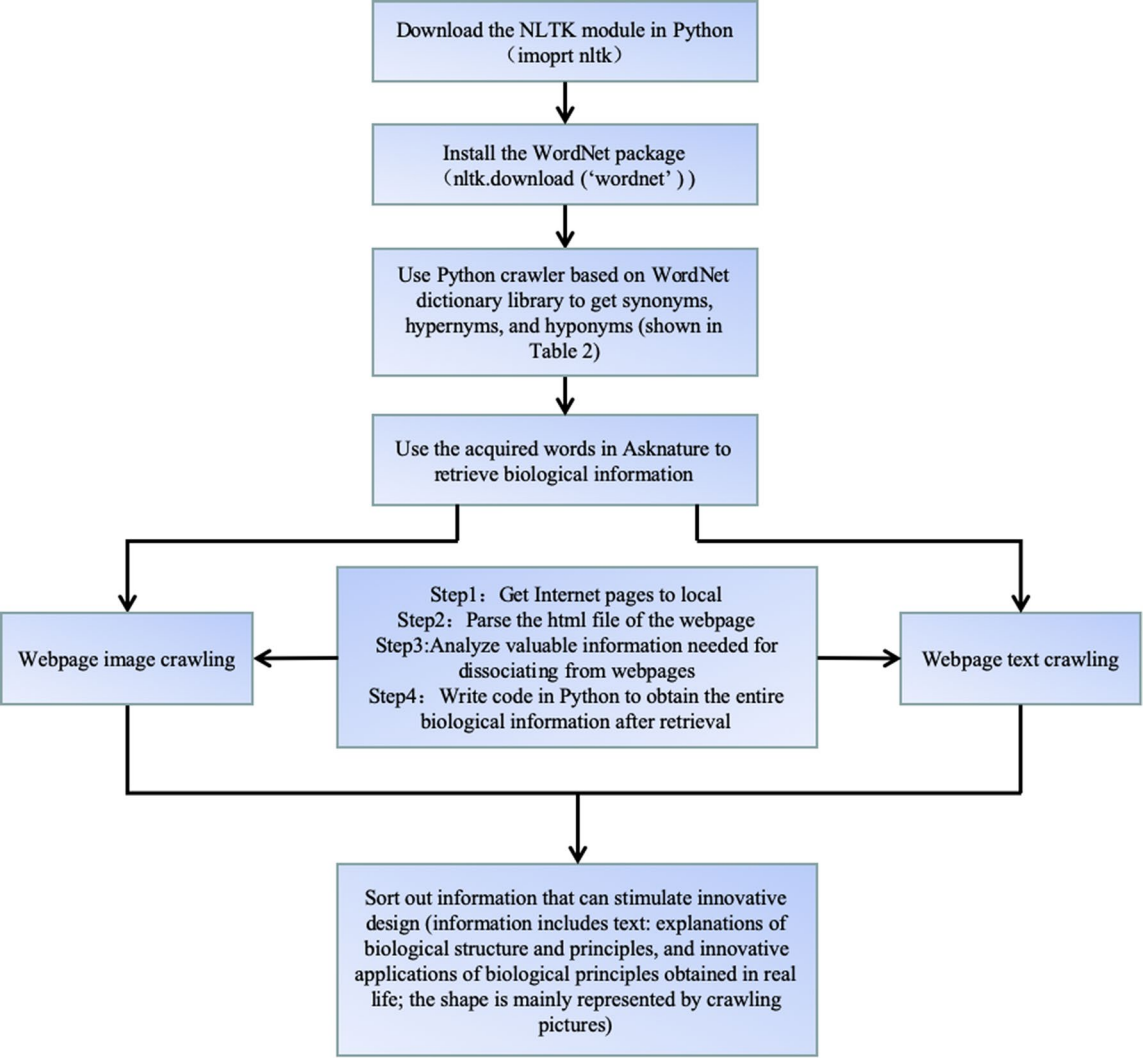
Flow chart of obtaining biological information based on Python.

The 20th century is the era of big data. Getting valuable information quickly and accurately on the Internet need to make full use of the advantages of computer programming languages. The fact is that people need to spend a lot of time in the design process to obtain the information. This paper uses the computer language Python to crawl biological information for saving a lot of time in the design process. Python can be used to filter the biological knowledge and information in biological databases. Of course, other computer languages can also handle it, but python is more powerful and rich in information processing. In this paper, biological information retrieved in AskNature mainly exists in the form of pictures and texts. The pictures include some biological shapes that can inspire design inspiration. The texts include some biological information that can inspire design inspiration, such as principles and structures. But usually a search term can retrieve many information links that may contain biological information used to inspire the design. We need to click on these links one by one to find useful information. But through the Python crawler, we can get all the pictures and texts information retrieved by a search term at one time, thus saving our time. The process of crawling web page information based on Python is shown in Fig. 6.

**Figure 7:**
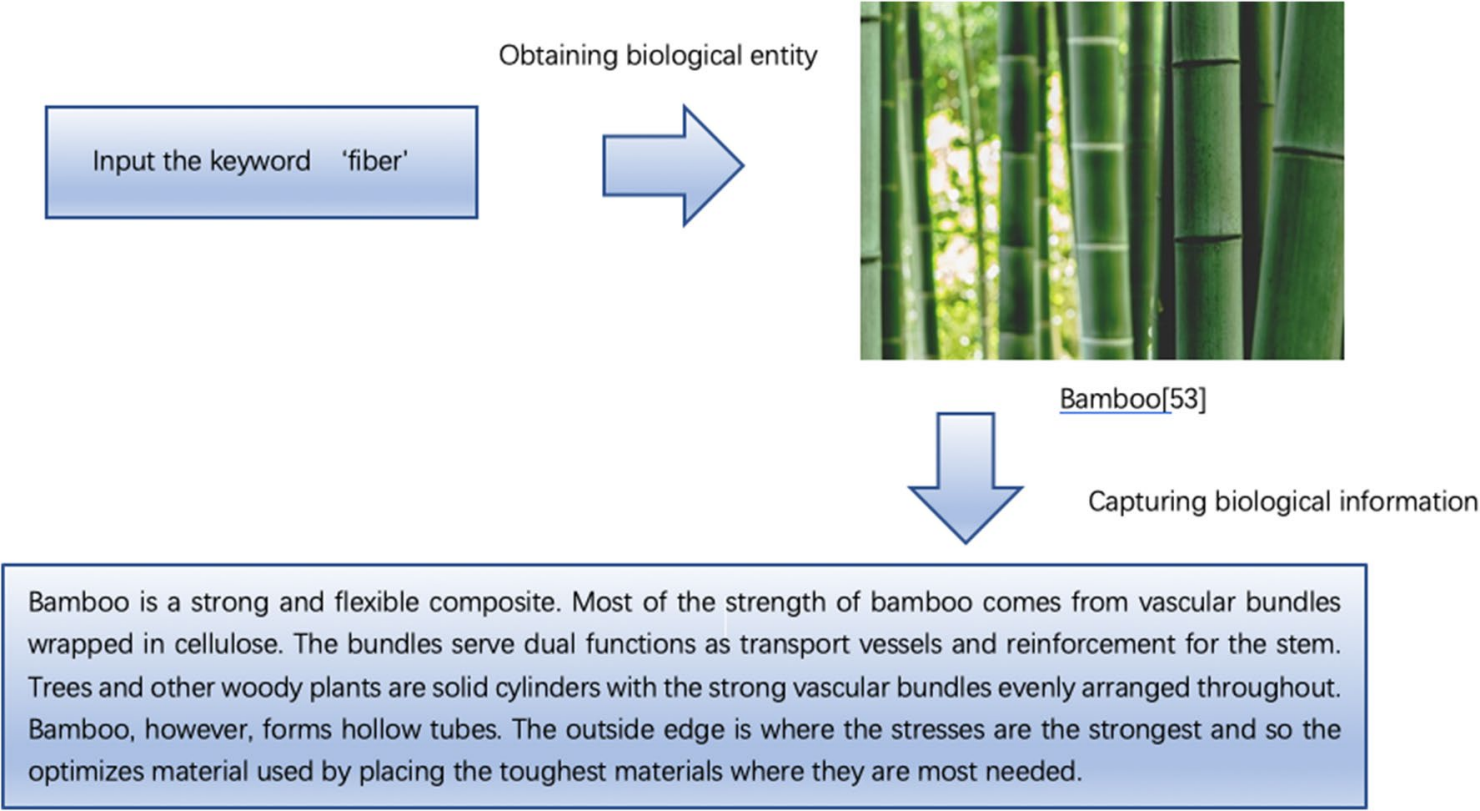
Process of capturing information in AskNature^54^.

Next, the filtered biological keyword ‘fiber’ is taken as an example and the biological information is obtained on the AskNature page based on Python programming. The flowchart is shown in Fig. 7.

#### Acquisition of biological knowledge

**Figure 8:**
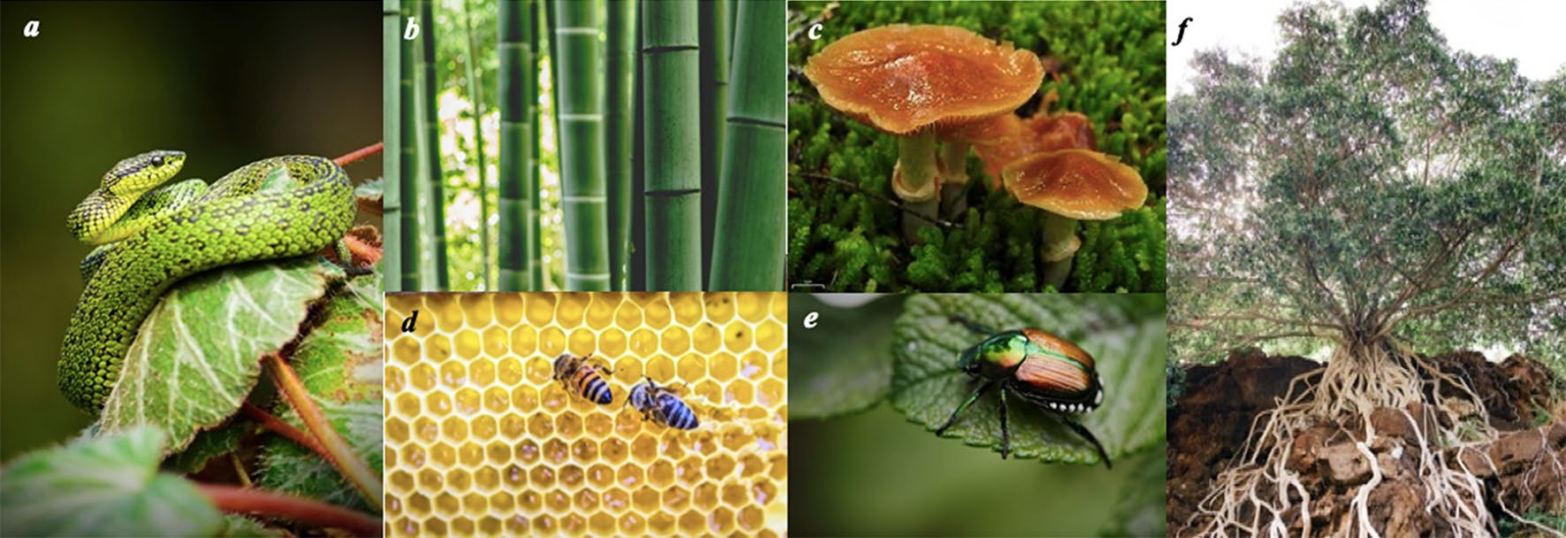
Biological entities retrieved by biological keywords. **(a**) Snake^55^; (**b**) Bamboo^53^; (**c**) Mushroom^56^; (**d**) Honey comb^57^; (**e**) Beetle^58^; (**f**) Root of a plant^59^. (All pictures are loyalty free).

Biological knowledge retrieved by keywords can not only stimulate the engineering designer, but also integrate the useful information in the biological field into the engineering design to achieve innovative design results. Based on the expanded biological keywords, searching on AskNature is first step in this paper, and then a Python program is used to crawl the relevant biological knowledge text, biological phenomenon and biological information, etc. The important features of biological objects are extracted and classified based on the function, the structure and shape features to aid the innovation of the winch. The acquired biological knowledge is shown on Table 4. Table 4 does not show all the biological information collected. Some conceives of using biological inspiration to design winch are also displayed on Table 4. Figure 8 shows some biological entities listed on Table 4.

**Table 4:** Biological knowledge triggering design inspiration.

Classification	Article name	Acquired biological knowledge^54^	Design inspiration
Function	Fiber arrangement is highly efficient	Most of the strength of bamboo comes from vascular bundles wrapped in cellulose. The bundles serve dual functions as transport vessels and reinforcement for the stem. Trees and other woody plants are solid cylinders with the strong vascular bundles evenly arranged throughout. Bamboo, however, forms hollow tubes. The outside edge is where the stresses are the strongest and so the optimizes material used by placing the toughest materials where they are most needed	This arrangement and distribution of bamboo inspired an idea whether a hollow structure can also be used in the winch drum, and then the hardest materials are gathered on the inner and outer walls of the tube, which can reduce weight and increase strength
	Structural composition provides strength in changing conditions	In the root tip or stem tip of a plant, the cell wall may be thinner in the immature stage, but when it grows to the mature stage, the cell wall becomes thicker, and lignin is incorporated into the structure. The cell itself has a strengthening effect on the overall strength of the plant. Parenchyma cells act like a pressurized container when fully hydrated. Mature cells, especially cells with thick cell walls, have their own strength even without water	The expansion and contraction caused by the complete hydration of the thin-walled wall is like a telescopic airbag. Is it possible to use such a telescopic airbag on the inner wall of the reel so that the contact area between the airbag and the rope is increased after the airbag is under pressure, thereby increasing the friction and achieving a non-slip effect?
Shape	Proteins reduce surface tension	Most mushrooms have an appendage on the surface, which is uneven	Is it possible to design a bionic covering layer with a spherical crown on the surface of the winch drum, so that the winch drum can prevent the rope from slipping during work?
	Scale shape enables limbless movement	Studies on a variety of different snake species have demonstrated that the friction generated by sliding depends on the direction of travel. Belly scales have small “micropatterns” that create arrays of v-shaped feathered trailing edges. The tips of these V-shapes point towards the tail of the snake and, in some species, they are raised at the tip. In this way, as the snake slides, the surface moves easily up and over the raised tips, but in reverse direction they act like the pawl of a ratchet, snagging the surface and resisting movement in the opposite direction. And snakes control movement by increasing friction on the surface of the skin and muscles	These V-shaped patterns on the surface of the snake can achieve the effect of preventing inversion. Can the ratchet pawl in this idea be used in the winch to prevent the movement of the cable when the winch rotates counterclockwise and cause damage to the people on the ship. In addition, the scales on the snake body are increased by Is it possible to apply this method of increasing friction to the outer surface of the drum to achieve a non-slip effect?
Structure	Honeycomb structure is space-efficient and strong	The honeycomb is composed of regular hexagonal beeswax cells, and the span is filled by adjusting the arrangement. The reason for the high compressive strength of the structure is that there are six short walls around each "tube"	Can the inner wall of the honeycomb hexagonal nesting structure be used in the first-round tube of the winch drum to enhance the ability of the drum to resist external pressure during work and reduce deformation?
	Layers create multihued appearance	The shell on the back of the beetle are curved structures, and both the shell and the beetle are hard material	Can the curved structure of the shell be applied to the pressure plate and the winch head in the winch to improve the pressure resistance and increase the service life of the winch head?

#### Triggering design inspiration

**Figure 9:**
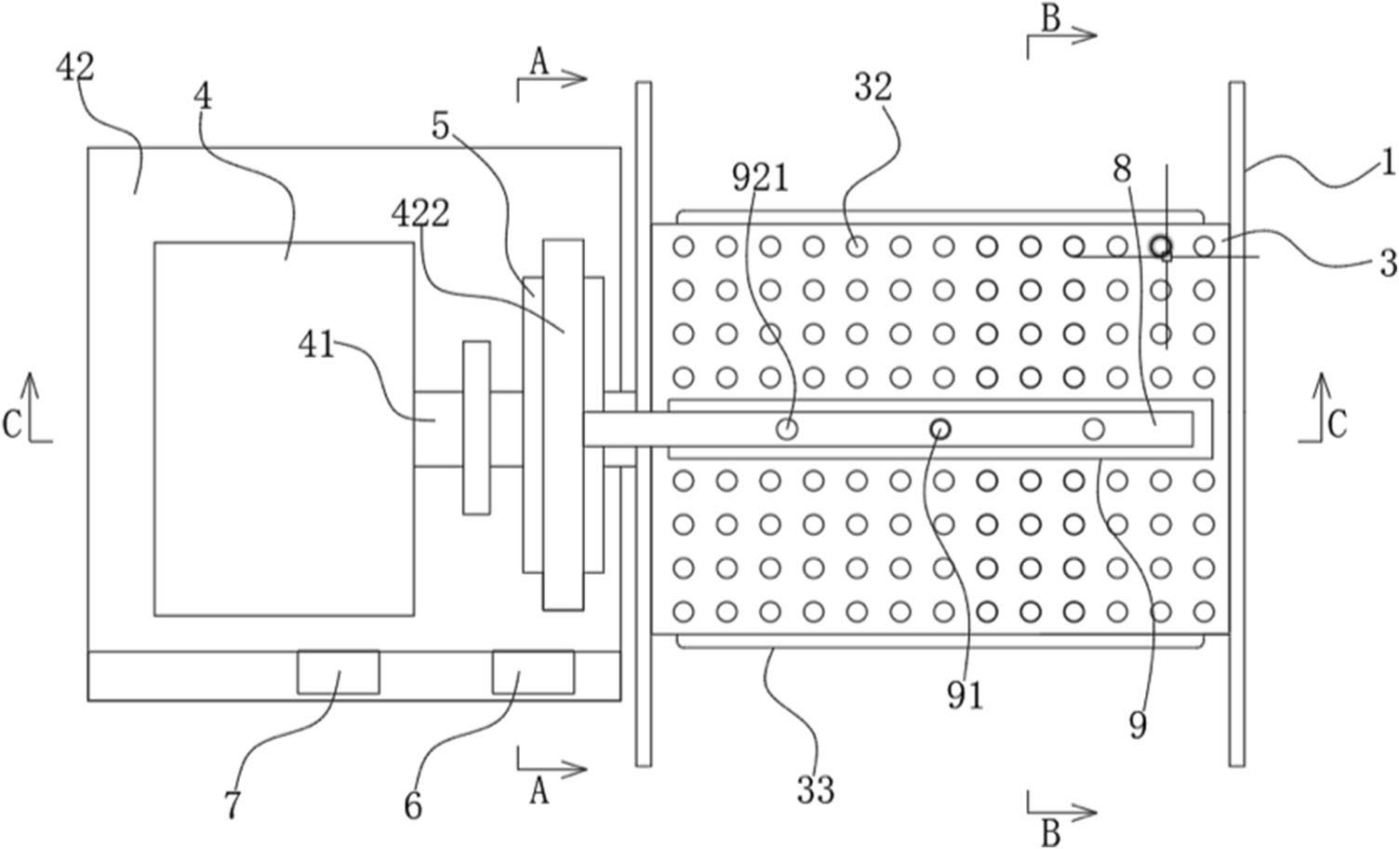
Structure diagram of electric winch^60^. 1—Side plate; 3—First round tube; 4—Drive motor; 5—Second round tube; 6—Controller; 7—Battery; 8—Fixing plate; 9—Curved pressure plate; 32—Rubber, bumps; 33—Telescopic airbag; 41—Rotary shaft; 42—Base; 422—Connecting rod; 91—Screw; 921—Sealing plate.

In life, biomimetic products are very common. Information and knowledge about biological appearance and other features can trigger designers to create novel designs. This kind of cross-domain information and knowledge is to a large extent the source of inspiration for future innovative design. This paper uses the analogy to transfer biological knowledge to engineering knowledge for solving the deficiencies of the existing marine winches. The novel marine winch designed is shown in Fig. 9, and the idea has been applied for an invention patent.

**Figure 10:**
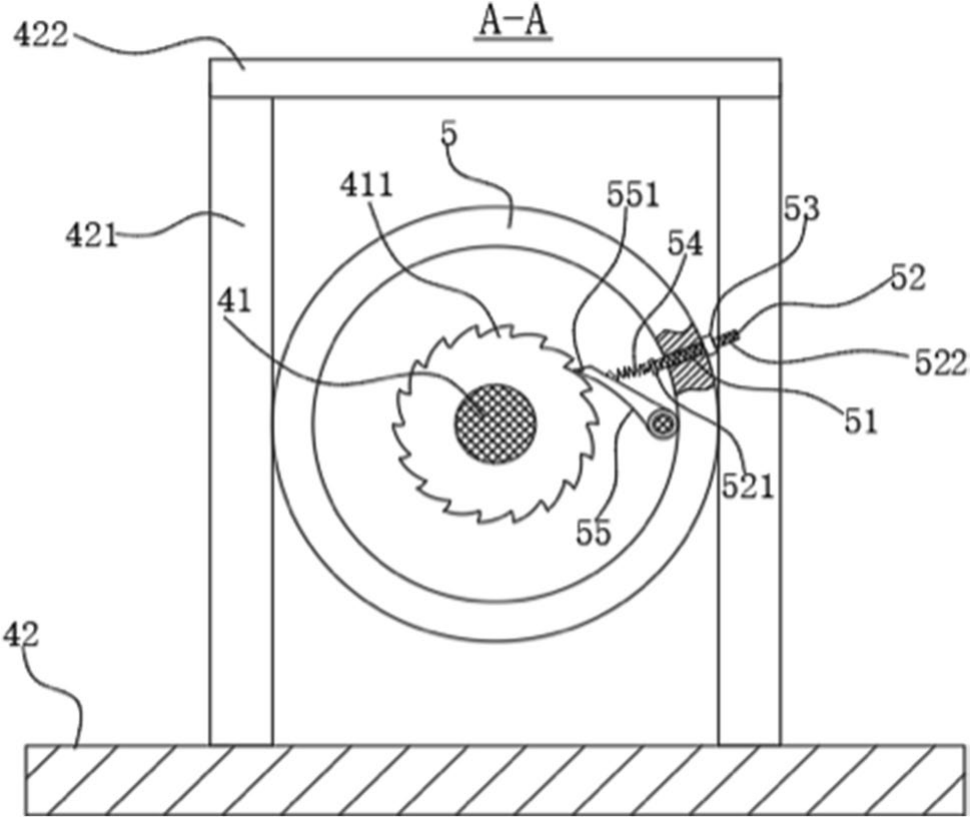
A-A section view^60^. 5—Second round tube; 41—Rotary shaft; 42—Base; 51—perforation; 52—Slider; 53—Second nut; 54—Compression spring; 55—pawl; 411—ratchet; 421—Support leg; 422—Connecting rod; 521—Limit plate; 522—External thread; 551—Pressure Sensor.

**Figure 11:**
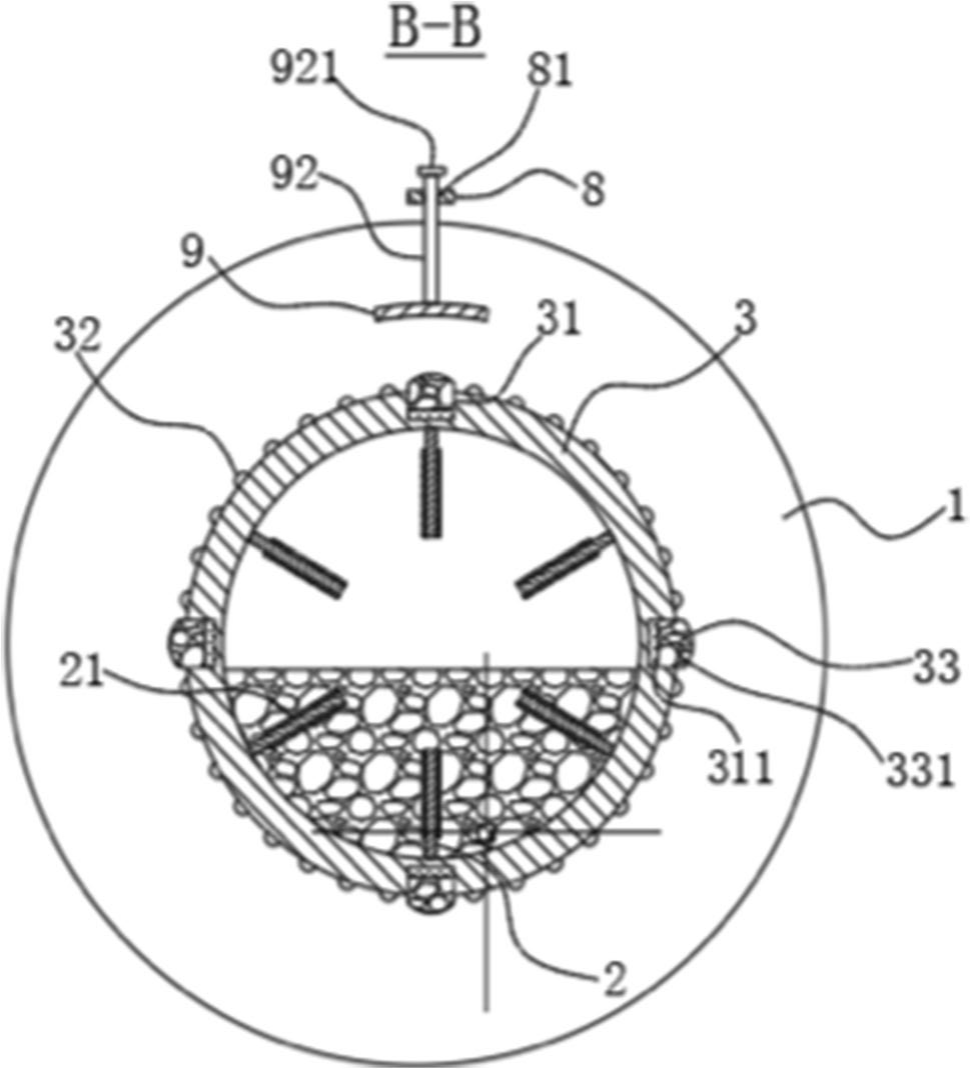
B-B section view^60^. 1—Side plate; 2—side panel; 3—First round tube; 8—Fixing plate; 9—Curved pressure plate; 21—Pressure generating sheet; 31—Groove; 32—Rubber bumps; 81—Through hole; 92—Vertical pole; 311—Electromagnet; 331—Magnetorheological fluid; 921—Sealing plate.

**Figure 12:**
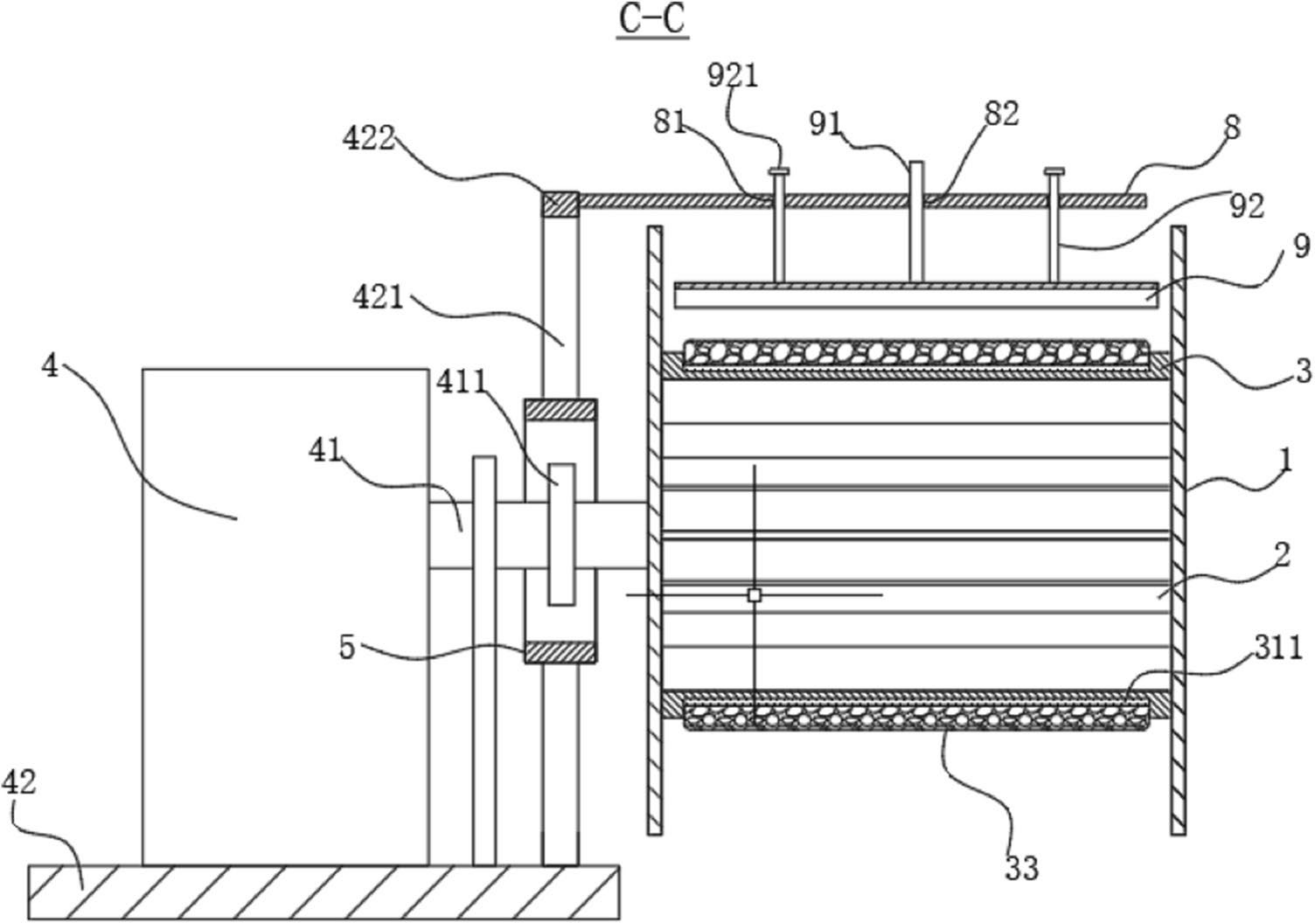
C-C section view^60^. 1—Side plate; 2—Side panel; 3—First round tube; 4—Drive motor; 5—Second round tube; 8—Fixing plate; 9—Curved pressure plate; 33—Rubber bumps; 41—Rotary shaft; 42—Base; 81—Through hole; 82-Threaded hole; 91—Screw; 92—Vertical pole; 311—Electromagnet; 411—ratchet; 421—Support leg; 422—Connecting rod; 921—Sealing plate.

The rubber bump in Fig. 9 is a biomimetic coating based on a layer of cover on the surface of a biological mushroom. The purpose is to prevent the wire rope from slipping. The ratchet and pawl mechanism in Fig. 10 is inspired by the micro-patterns on the abdomen of the snake. It can prevent the shaft from turning upside down. The surface of the snake can easily move up and over the protruding tip during the sliding process. In turn, they hook the surface like the pawls of a ratchet and prevent movement in the opposite direction. The retractable airbag in Fig. 11 is designed based on the hydration of the plant cell wall. The purpose is to increase the contact area between the steel wire rope and the first-round tube when the telescopic airbag is compressed and deformed, thereby increasing the friction. The curved pressure plate in Fig. 12 is designed according to the shape and structure of the shell and the back of the beetle. The purpose is to increase the force area and improve the pressure resistance. The outward round tube uses a hollow tube and the inner wall reinforcement plate is designed based on bamboo. Most of the strength comes from the arrangement and structure of vascular bundles wrapped in cellulose fibers.

The above solutions are conceived based on the acquired biological knowledge. The specific details are shown in Table 5:

**Table 5:** Design choices and details.

Selected organism or part	Detail
Bamboo	The shape of bamboo is cylindrical and similar to that of the reel in the winch. The arrangement of bamboo fiber bundles is a ring-shaped mesh hollow structure, which can be realized on the inner and outer walls of the reel. The hollow structure of the first-round tube can reduce the overall weight of the winch and is convenient for handling. The inner and outer wall structures can improve the structural strength and avoid deformation under compression
Rhizomes and pointed stems of plants	The hydration of rhizomes and tip stems can cause cell walls to expand and contract, similar to retractable air sacs. This kind of bionic airbag structure is added into the groove, the steel wire rope is in contact with the first round tube, the airbag is deformed under pressure, the contact area between the first round tube and the steel wire rope is increased, and the friction force can be increased
Mushroom	There are many covering layers on the mushroom surface, and the covering layer is designed on the first-round tube with rubber bumps to prevent the wire rope from slipping
Snake's abdomen scales	Abdomen scales are like an inverted V structure. This inverted V structure is designed on the rotating shaft with a ratchet and pawl. When the drive motor suddenly loses power, the weight loses the pulling force and falls, and the rotating shaft is impacted in one direction. At this time, the pawl restricts the ratchet from turning Rotate to avoid accidental falling of heavy objects
Honeycomb structure	The hexagonal structure of the honeycomb is designed in a bionic design on the inner wall reinforcing plate of the reel to improve the strength of the first-round tube
The shell on the back of the Beetle	The shell of the beetle is mostly arc-shaped. The arc-shaped structure is used as the arc-shaped pressure plate of the winch. When the winch drags the heavy object, the steel wire rope releases the specified length, and the arc-shaped pressure plate is moved down and pressed by the lifting structure. The steel wire rope of the first-round tube avoids the relative sliding of the steel wire rope and improves the working stability of the winch

## Discussion

### Biology dictionary

The primary problem of stimulating design inspiration with the help of acquired biological knowledge is how to determine keywords. The different keywords can retrieve different biological information. This paper uses three biological backgrounds, namely Biology-online.org, Henderson’s dictionary of Biology, and dict.cnki.net, to obtain biological keywords. After using, the advantages and disadvantages of three ways are found as following:

- The keywords extracted from the dictionary module of Biology-online.org need to be analyzed to obtain more intuitive biological keywords in the retrieved articles. Therefore, extracting keywords in the article will increase the time consumption.
- Henderson’s dictionary of Biology text dictionary can quickly locate the searched word, but this biological dictionary is a knowledge explanation of the searched word. Therefore, the efficiency is not high.
- Dict.cnki.net contains information in various fields. Users only need to select biology after retrieving content related to biology only, and the biological information retrieved is only a biological text related to the search term information. Therefore, it greatly helps users save time, and the extracted keywords will be more accurate and closely related to biological themes.

### Comparison of biological keyword expansion methods

In order to obtain more comprehensive biological knowledge from AskNature, this article uses two methods to expand biological search terms. The biological terms are expanded in Thesaurus and in Python based on the WordNet database. Through practice, the advantages and disadvantages of the two methods are found as follows:

- The advantage of the expansion in Python based on WordNet database is that not only it can crawl synonyms, but also crawl the hypernym and hyponym with the same part of speech as the search term. The whole the process is automated. The disadvantage is that fewer words are obtained and the process is more complicated.
- The advantages of synonym retrieval from biological keywords in Thesaurus are that the process is easier and can obtain more words. Some words crawled by Python will also be included.

Using both expansion methods at the same time will complement each other. Keywords obtained by the two expansion methods are shown on Table 6.

**Table 6:** Expansion method comparison.

Input word	Retrieve Webpages for expanded words (Thesaurus Webpage)	Python automatically obtains expanded words (WordNet database)
Shell	Carapace, frame, integument, pod, skeleton, case, chassis, crust, frame, hull, husk, nut, pericarp, plastorn, scale, shard, shuck, skin	Carapace, palte, ammunition, shotgun-shell, shrapnel
Reduction	Contraction, rebate, debasement, cut, cutback, devaluation, discount, abatement, attrition, cortailment, minimization, diminution, degradation, decrement, shrinkage, subjection, subtraction, subdual, markdown	Decrease, change-of-magnitude, amortization, contraction, depreciation, lowering, shortening, shrinking, weakening, subtraction, depletion, easing, discount, cutback, conquest, declassification, rollback, reverse, de-escalation, deflation, extenuation, discount
Fiber	Grain, thread, tissue, cilia, cord, fibril, filament, grit, hair, shred, staple, string, strip, tendril, tooth, vein, warp, web, woof, footlet	Material, roughage, character, Bassine, bristle, coir, string, raveling, oakum

The method proposed in this paper has similarities and differences with the method provided by Lenau^61^. The similarity is that we have supplemented and refined the keywords. Lenau generated synonyms for the keywords in the list based on the thesaurus function in the online Google Docs of Encyclopedia Britannica. This article uses Python programming to automatically obtain synonyms based on the WordNet database. The difference is that Lenau expanded keywords for the first time in AskNature to obtain more possible keywords and generate more words at the same time in the continuous progress of the search stage. This article uses keywords based on different biological backgrounds, such as biology and biological dictionaries, to expand the purposeful keywords that are closer to biology. Finally, we can get biological knowledge in AskNature based on these keywords. In contrast, the advantages of the method proposed in this article are to simplify the biological keywords, expand the keywords after narrowing the scope of keywords, and finally obtain the required biological knowledge and information in AskNature automatically. Therefore, this method can not only shorten the acquisition time, but also ensure the relevance of biological and engineering keywords under the condition of expanding biological knowledge.

In addition, the method proposed in this paper has similarities and differences with the method provided by Kaiser^19,20^. The similarity is that we all use the function of the product as engineering keywords and WordNet to expand search space. The difference is that the papers of Kaiser mainly include three steps, namely, selecting engineering terminology, obtaining search terms, and acquiring biological knowledge. But this paper includes five steps, namely, selecting engineering terminology, obtaining biological keywords, expanding biological keywords, filtering biological keywords and acquiring biological knowledge.

There are some differences in these steps.

1. *Selecting engineering terminology* Kaiser^19,20^ chose function, property and environment of the product as the engineering terminology. This paper selects the product’s purpose function, means function, and key components as engineering terminology according to the type of knowledge in the product^48^. The purpose functions are related to the performance of the product, such as the cost, volume, requirement, etc. For example, ‘reducing vibration’. The means functions involve the methods and means to improve the technical, economic, or social aspects of a product, such as ‘isolate’. It means to reduce vibration by isolating objects. In addition, the function, shape, behavior and other features are hidden in the names of some parts, such as ‘elastic ring’ and ‘clamping devices’. Among them, the name of the parts is easily overlooked. Choosing these engineering terminologies can more accurately reflect the needs of engineers.
2. *Obtaining search terms* Kaiser^19,20^ obtained the search terms with the help of WordNet. This paper uses some database, namely dic.cnki.net, Biology-online.org Webpage and Biology-online.org dictionary module to obtain biological keywords.
3. *Expanding biological keywords* There is no such step in the Kaiser^19,20^. This paper uses two ways to expand biological keywords. One way is to use Thesaurus Webpages to expand biological keywords, and the other way is to use WordNet to expand biological keywords with the help of python programming. So, the more biological keywords can be obtained, and the same keywords obtained through these ways can be preferentially used for retrieval.
4. *Filtering biological keywords* There is no such step in the Kaiser^19,20^. This paper uses two methods to filter biological keywords. One method is to filter out some redundant words. The second method is to filter out different forms of the same word. These processes can be easily completed through programming. Filtering out some words can reduce the number of useless biological keywords, thereby reducing retrieval time.
5. *Acquiring biological information* Kaiser^19,20^ acquired biological knowledge from the PubMed. This paper acquires the biological knowledge from AskNature with the help of Python crawling programming.

The proposed method uses different and easily overlooked but important engineering terminologies, expands and filters biological terminologies in multiple ways, thereby it can expand the search space of terminologies, increase the possibility of obtaining more valuable biological terminologies, and acquire more biological knowledge quickly by using Python crawling technology.

### Comparative experiment

Comparison with Chen^15^, the same point is that this paper and Chen^15^ are both based on WordNet database to obtain synonyms to retrieve biological information and provide auxiliary innovative design ideas for engineering design. The differences between the two methods is that in the process of obtaining biological terminology. In this paper, based on the feature of engineering design products, some engineering words, such as function, purpose, shape and other engineering words, are selected. Some database, namely dic.cnki.net, Biology-online.org Webpage and Biology-online.org dictionary module are used to obtain biological terminologies. Chen decomposes engineering requirements and structure FCW, calculates FCW's ECI (Engineering Correlation Intension), BCI (Biological Correlation Intension), and CCI (Composite Correlation Intension) values, and then pushes keywords through ranking. The potential words can be found and commended based on the calculated value.

In order to observe the effect of using the presented method to acquire biological knowledge, we use the phrase ‘transfer electric energy’ in Chen^15^ to compare the amount of biological information obtained with this method and the method in Chen^15^.

According to the proposed method, the phrase is split and expanded by two ways. The expanded biological terminologies is shown in Table 7.

**Table 7:** Biological terminologies expanded by the two ways in the proposed method.

Keywords after splitting	The number of search terms after expanding	
	Based on Thesaurus	Based on WordNet
Transfer	18	12
Electricity	4	11
Energy	54	13

When two ways are used to expand biological terminology, the expanded words generated by one way may also exist in the expanded words generated by the other way, and these repeated words will be preferentially used for retrieval. Table 8 is the comparison of the number of search terms and the number of retrieved biological information between Chen^15^ and the proposed method. (ST: Search Term, NPBI: Number of pieces of biological information retrieved)

**Table 8:** The number of pieces of biological information retrieved by the two methods.

	ST	NPBI
Chen^15^	Transfer electron	40
	Transfer charge	28
	Transfer potential	16
	Shift electron	1
	Shift potential	3
	Pump electron	12
	Transfer field	9
	Displace electron	9
	Shift field	2
	Migrate charge	13
This Paper	Transfer efficiency	1
	Transfer electron	11
	Transfer intensity	1
	Conduction power	36
	Substitution power	35
	Conduction energy	2
	Movement energy	3
	Movement power	75
	Transport electronics	19
	Change electron	23
	Delivery charge	35

**Figure 13:**
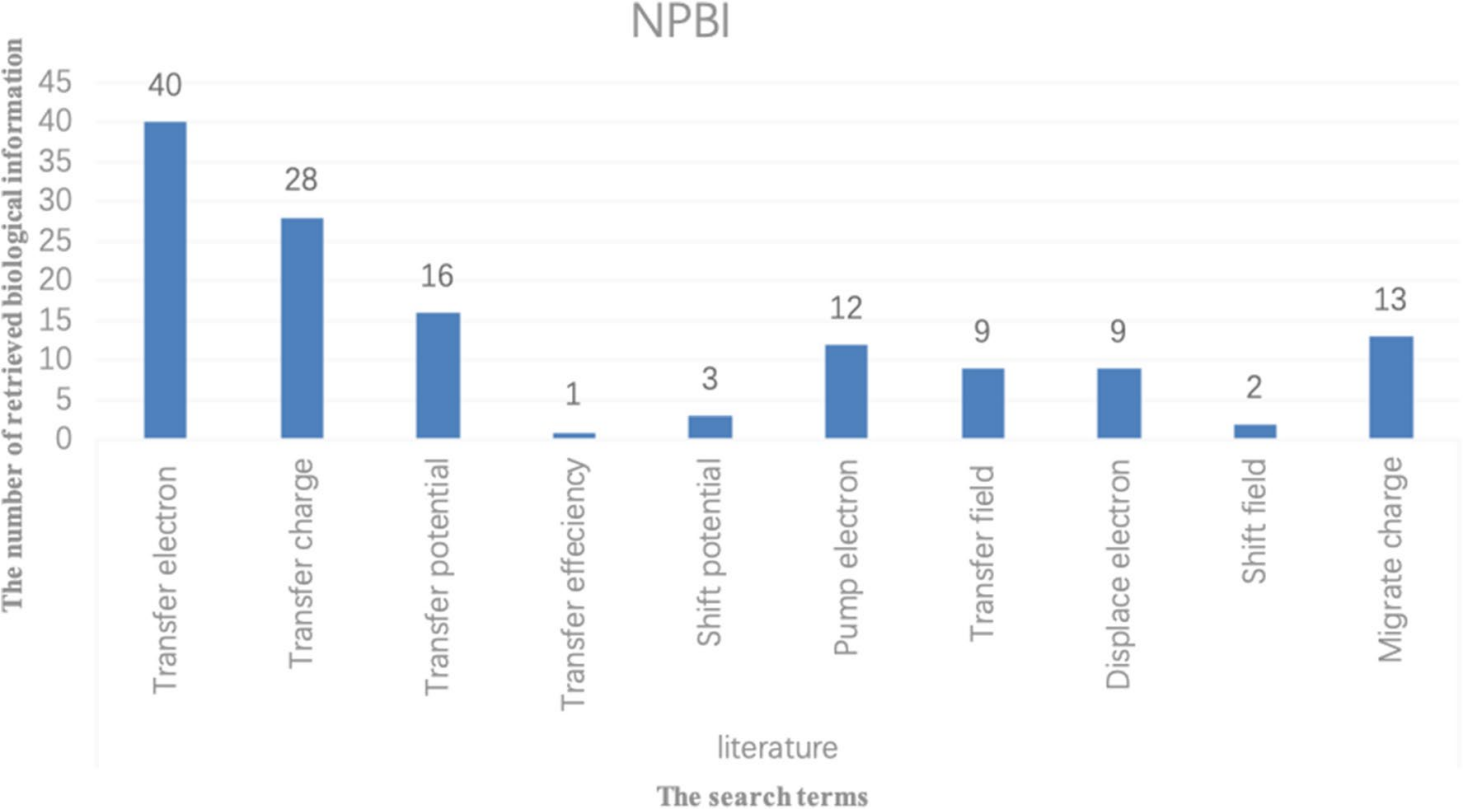
The number of biological information retrieved by Chen^15^.

**Figure 14:**
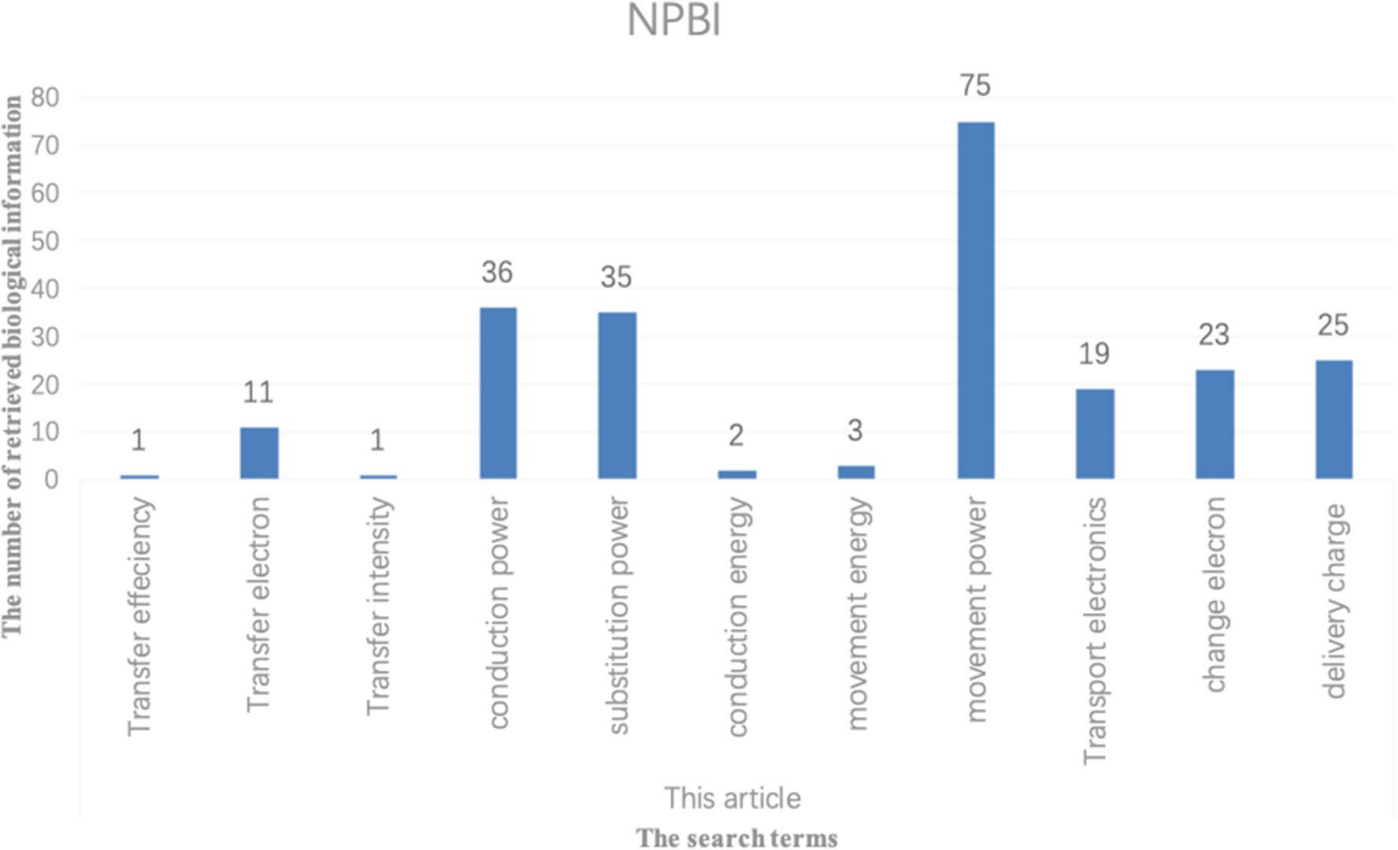
The number of biological information retrieved by the proposed method.

It can be found from Table 8 and Figs. 13 and 14 that different biological terms can be obtained by the proposed method and Chen^15^, and thus different biological information can be obtained. Although the number of search terms used by the two methods is similar, the biological information retrieved is quite different. This difference is mainly due to the two ways of expanding biological keyword used in this article and obtaining biological keywords from multiple databases.

## Conclusions

In the design process, if there is no stimulation from external information, the designer's development and innovation ideas will be increasingly limited to traditional thinking. Biological knowledge is an important source to stimulate design inspiration. But without biological knowledge, it is difficult for people in different fields to obtain the biological information required. Therefore, acquiring biological knowledge is a key step in the innovation process. The method proposed in this paper can not only stimulate designers' inspiration, but additionally has lower requirements for designers' interdisciplinary knowledge than other automatic acquisition methods such as algorithms and natural language analysis. At the same time, in the process of acquiring biological knowledge, Python language is used to expand keywords and capture biological information, which expands the space for acquiring biological knowledge and shortening the time of biological information retrieval.

Using Python language to expand biological keywords through biological dictionaries and biological databases can greatly increase the number of keywords, but at the same time, the expanded biological keywords will have more word polymorphisms and word repetitions, etc., manual filtering will take a lot of time. Therefore, the next step of this work is to solve the problem of automatic selection of expanded words. In addition, there are different types of biological websites on the Internet. How to use multiple biological websites to obtain complementary biological knowledge for analogy stimulation and obtain new the inspirational design is also a very interesting and worthy research question.

## Data Availability

All data generated or analyzed during this study are included in this published article.
